# Physician Perceptions on the Use of Antibiotics and Probiotics in Adults: An International Survey in the Asia-Pacific Area

**DOI:** 10.3389/fcimb.2021.722700

**Published:** 2021-10-19

**Authors:** Uday C. Ghoshal, Kok-Ann Gwee, Gerald Holtmann, Yanmei Li, Soo Jung Park, Marcellus Simadibrata, Kentaro Sugano, Henry Cohen, Eamonn M. M. Quigley

**Affiliations:** ^1^Department of Gastroenterology, Sanjay Gandhi Postgraduate Institute of Medical Sciences, Lucknow, India; ^2^Department of Medicine, Yong Loo Lin School of Medicine, National University of Singapore, and Gleneagles Hospital, Singapore, Singapore; ^3^Faculty of Medicine and Biomedical Sciences, University of Queensland, Brisbane, QLD, Australia; ^4^Department of Gastroenterology and Hepatology, Translational Research Institute, Princess Alexandra Hospital, Brisbane, QLD, Australia; ^5^Department of Gastroenterology, China-Japan Friendship Hospital, Beijing, China; ^6^Department of Internal Medicine and Institute of Gastroenterology, Yonsei University College of Medicine, Seoul, South Korea; ^7^Department of Internal Medicine, University of Indonesia, Jakarta, Indonesia; ^8^Department of Gastroenterology, Jichi Medical University, Shimotsuke, Japan; ^9^Department of Gastroenterology, Universidad de la República, Montevideo, Uruguay; ^10^Gastroenterology and Hepatology, Lynda K. and David M. Underwood Center for Digestive Disorders, Houston Methodist Hospital and Weill Cornell Medical College, Houston, TX, United States

**Keywords:** antibiotic-associated diarrhea, probiotics, antibiotic resistance, antibiotic prescriptions, microbiota

## Abstract

**Background and Aims:**

The over-prescription of antibiotics is thought to represent a major threat to public health worldwide and is more frequently observed in some low- and middle-income countries. In the Asia-Pacific region, economic development, health care organization and population demographics are very heterogenous. The objective of this survey was to investigate antibiotic use and probiotic co-prescription among adult patients in this area.

**Methods:**

An online survey of physicians from seven countries of the Asia-Pacific region (Australia, Japan, Indonesia, India, China, Singapore and South Korea) was performed in 2018. The questionnaire explored current practices of physicians concerning antibiotics and probiotics and factors related to prescribing decisions.

**Results:**

A total of 387 general practitioners and 350 gastroenterologists completed the questionnaire. Physicians in Australia, Japan and South-Korea were low prescribers of antibiotics (11% to 19% of visits resulted in an antibiotic prescription), while physicians in Indonesia, India, China and Singapore were high prescribers (41% to 61%). A large majority (85%) of physicians agreed that antibiotics disrupted intestinal microbiota. The rates of co-prescription of probiotics varied from 16% in Japan to 39% in Singapore (overall, 27%). Conditions considered by physicians to be prevented by probiotics were mostly antibiotic-associated diarrhea (62%) and *Clostridium difficile* colitis (43%).

**Conclusions:**

Rates of probiotic co-prescription remain low in many countries although the negative effects of antibiotics on the gut microbiota and the benefits of co-prescribing probiotics are generally known.

## Introduction

Between 2000 and 2015, the worldwide consumption of antibiotics increased by 65% (WHO, 2019). This increase was mainly driven by low- and middle-income countries where consumption was positively correlated to the gross domestic product per capita (Klein et al., 2018). The global consumption of antibiotics is a major factor in the emergence of bacterial strains resistant to antibiotics. As a consequence, the efficacy of commonly used antibiotics is threatened (Tacconeli et al., 2018).

The solution to the “antibiotic resistance crisis” must involve a reduction in the inappropriate use of antibiotics in both human and veterinary medicine, appropriate use of antibiotics where indicated and the development of new antibiotics (Rossolini et al., 2014; Martens and Demain, 2017). However, the burden of infectious diseases persists in low- and middle-income countries and global surveillance must consider the persistence of inequities in drug access (Klein et al., 2018).

The gut microbiota has important roles in human physiology, metabolism and immunity (Duan et al., 2020). The administration of oral antibiotics is associated with gut dysbiosis which includes a loss of diversity of microbiota, changes in the distribution of some taxa and expansion of antibiotic-resistant bacteria. Some beneficial commensal bacteria such as *Bifidobacterium* and *Lactobacillus* are depleted after oral treatment by beta-lactam, glycopeptide and macrolide antibiotics. In addition, antibiotic treatment disrupts the different components (secretory, physical and immunological) of the intestinal barrier (Duan et al., 2020). Antibiotic-associated diarrhea (AAD) is the consequence of the disturbance of the composition of the intestinal microbiota and the alteration of its protective function, thus leading to opportunistic infections, mucosal immune disorders and diarrheal disease (Duan et al., 2020). Most often, cases of mild AAD are related to functional disorders of intestinal carbohydrate or bile acid metabolism; other causes of AAD are allergic and toxic effects of antibiotics on gut mucosa or effects on intestinal motility (McFarland, 2008). The prevalence of AAD ranges from 5% to 30% and occurs either early at the onset of treatment or up to two months after the end of treatment (McFarland, 2008).

*Clostridium difficile* (or *Clostridiodes difficile* according to the new terminology) is a bacterium which is the main cause of hospital-associated infectious diarrhea (Shah et al., 2010). The incidence of *C. difficile* infection ranges from 2.45 to 7.5 per 10,000 patient days or 9 to 80 per 10,000 patient admissions (Johnston et al., 2018). Although diarrhea is its most frequent presentation, *C. difficile* infection can lead to pseudomembranous colitis, toxic megacolon or death; the mortality associated to *C. difficile* infection ranges from 5% to 10% The most important risk factor is the exposure to antibiotics which disrupts the gut microbiota thus allowing *C. difficile* to proliferate (Johnston et al., 2018).

Antibiotic-induced disturbances in the composition and functions of the intact gut microbiome have been also implicated in the pathogenesis of inflammatory bowel disease, obesity, colon cancer and other disorders (Yoon and Yoon, 2018).

In order to restore the equilibrium of the intestinal microbiota, the administration of numerous strains of living organisms (probiotics) has been proposed (*Lactobacillus acidophilus*, *Lacticaseibacillus rhamnosus* GG, *Lactobacillus delbrueckii* subsp. *bulgaricus*, *Bifidobacterium bifidum*, *Bifidobacterium longum*, *Enterococcus faecium*, *Streptococcus thermophilus* or *Saccharomyces boulardii* (Barbut and Meynard, 2002; Guarner et al., 2017). Meta-analyses have indicated that the co-administration of probiotic strains reduced the risk of AAD (relative risk 0.43 to 0.61 in comparison to control) (McFarland, 2006; Pattani et al., 2013; Szajewska and Kolodziej, 2015). For example, a recent meta-analysis reported that the administration of *S. boulardii* reduced the risk of AAD in patients treated with antibiotics from 18.7% with placebo or no treatment to 8.5% (Szajewska and Kolodziej, 2015). A meta-analysis showed that probiotic prophylaxis could prevent *C. difficile* infection, particularly in hospitalized patients and in patients treated with two or more antibiotics (Johnston et al., 2018). Nevertheless, some concerns have been raised about the potential of some bacterial (Imperial and Ibana, 2016), but not yeast-based probiotics (Czerucka et al., 2007), to convey antibiotic resistance genes through horizontal gene transfer.

The key measures to prevent AAD are to limit use of antibiotics and to promote effective hygiene measures. In addition, probiotics have proven to be useful in preventing AAD. Recently, the working group of the European Society for Paediatric Gastroenterology Hepatology and Nutrition (ESPGHAN) recommended probiotics for preventing AAD and *C. difficile*-associated diarrhea (Szajewska et al., 2016). Similarly, an Asian expert consensus recommended the administration of a probiotic with an antibiotic in order to reduce the frequency of AAD and *C. difficile*-associated diarrhea (Ghoshal et al., 2018).

In the Asia-Pacific region, population demographics, levels of economic development, organization and delivery of health care and public health policies vary considerably from country to country (World Health Organization, Regional Office for South-East Asia‎, 2008). Our objective, therefore, was to investigate current physician practices across this diverse Asia-Pacific region relating to antibiotic use and probiotics co-prescription in adult patients.

## Materials and Methods

An online survey (complete survey provided in **Supplementary Materials as Table 1**) was conducted among physicians [general practitioners (GPs) and gastroenterologists] in seven countries of this region (Australia, Japan, Indonesia, India, China, Singapore and South Korea) in August and September 2018. The World Gastroenterology Organization (www.worldgastroenterology.org) endorsed the questionnaire. This study was based on the opinions of physicians collected in an online survey and no ethics committee approval was required.

The main objectives of the survey were to assess (i) current practice in terms of antibiotic use in adults in order to understand what factors determined prescribing patterns, (ii) rates of probiotic co-prescription and (iii) to evaluate the perceived benefits of probiotics.

The on-line questionnaire was divided into two parts, one for primary care providers and one for gastroenterologists. The questionnaire included questions on antibiotic and probiotic prescription and also asked the respondents to provide their approach to two real-life clinical situations (a 2-day history of acute respiratory symptoms and fever in an adult for GPs and a 2-day history of acute infectious diarrhea and fever in an adult for gastroenterologists).

The survey was primarily web-based, but for some countries (Indonesia, India, Singapore, South Korea), a mixed data collection methodology was necessary in order to achieve recruitment objectives. The average duration for completing the questionnaire was 10 min. A compensation for participation was offered to physicians (except Chinese physicians and a fraction of Indian physicians who were not compensated).

The questionnaire was translated and physicians answered in the following languages: English for Australia, India and Singapore and in local languages for Japan, Indonesia, China and South Korea.

The sample size was calculated in order to achieve statistical robustness and reliable analysis of the total sample and subgroups (countries and specialties). A total of 737 physicians were enrolled including 387 GPs and 350 gastroenterologists in Australia (50 GPs/50 gastroenterologists), Japan (50/50), Indonesia (50/40), India (70/70), China (70/70), Singapore (47/20) and South Korea (50/50).

The analysis was mainly descriptive with categorical variables summarized by frequencies and proportions and continuous variables summarized by means and ranges. Data were presented overall and after comparisons between countries or specialties. Formal comparative tests of significance were performed with a 95% confidence interval on the following criteria: within each specialty: total vs country; within each country: gastroenterologists vs GPs - using a standard normal table. An overall weighting was applied for total results (sum of countries and/or specialties) to provide the same weight in results regardless of the number of physicians who answered the question. For results by country and specialty (no sum), results were unweighted. All statistical tests were two-sided and differences regarded as significant at the level of 0.05.

## Results

### Profiles of Physicians

The demographics of the surveyed physicians varied according to countries. In some countries, physicians were mainly male: Japan (99%), India (92%) and South Korea (87%); in the other countries, sex ratios were more balanced (% male: China 54%, Australia 77%, Singapore 61%, Indonesia 68%). The most frequent age class of physicians was 45–59 years, with the exception of China and Singapore, where they were predominantly younger than 45 years. The number of years of practice ranged from 9 years in China to 25 years in Japan.

The vast majority of physicians had a private practice (from 65% in Indonesia to 97% in India), except in China (88% had a public practice). Working time was mainly spent in hospital in all countries (from 69% in Singapore to 92% in Japan), except in Australia (74% in office). Academic activity was frequently reported by physicians in Australia (67%) and China (89%), but was low in Japan (13%) and India (9%).

The primary practice location was largely urban in Australia (88%), Indonesia (100%), India (100%), China (92%), Singapore (97%) and South Korea (77%); in Japan, 52% of physicians worked in rural areas. The average number of patients seen per month varied considerably, from 240 in China to 780 in South Korea.

### Prescription of Antibiotics and Perceived Risks

**Figure 1:**
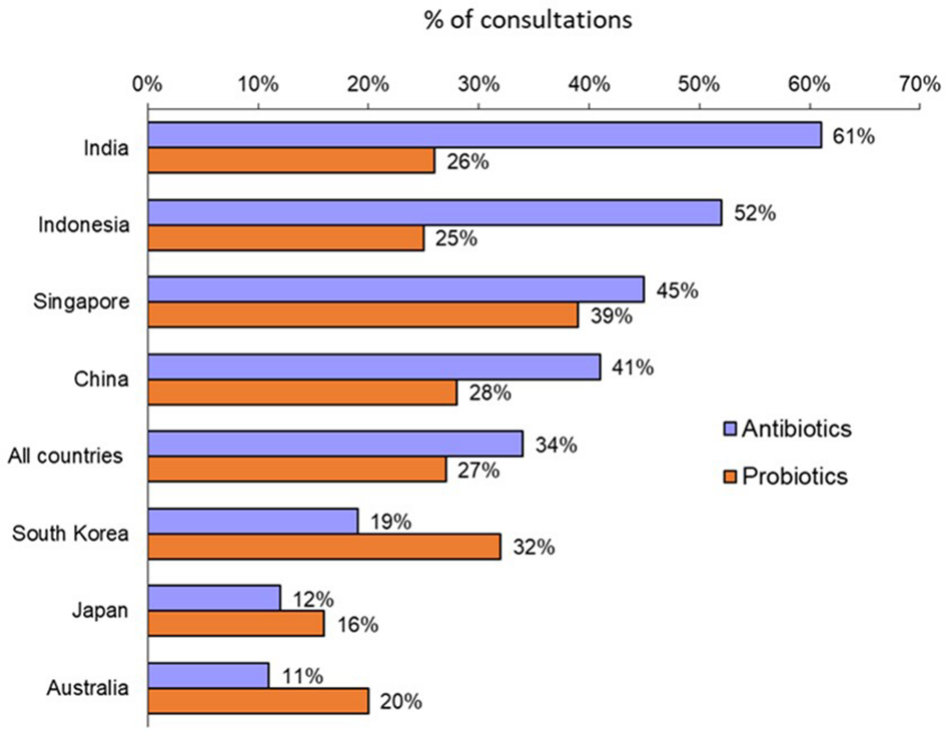
Rates of antibiotic prescription and probiotic co-prescription for all adult consultations (Question B1: “*Over the past year, what was the approximate percentage of all your adult consultations that led to antibiotic prescription?*”; Question B7: “*For what percentage of adult patients receiving antibiotics do you co-prescribe probiotics?*”).

Overall, a consultation with an adult patient led to a prescription for an antibiotic in 34% of cases with large variations according to countries (**Figure 1**). Physicians in Australia, Japan and South Korea were low prescribers of antibiotics (11% to 19% of physician visits led to the prescription of an antibiotic), while physicians in Indonesia, India, China and Singapore had higher rates (from 41% to 61%).

Rates of antibiotic prescription were patient age-dependent rising from 12% for young adults (18–35 years of age) to 46% for middle-aged adults (36–55 years) and 42% for older adults (> 55 years). Indeed, the highest rates of antibiotic prescription were for older adults in Japan (85%) and South Korea (74%).

Physicians ranked the following factors that restrained them from prescribing antibiotics to an adult in order of importance: risk of antibiotic resistance (34%), possible side effects (27%), lack of efficacy (26%) and concerns regarding an alteration of intestinal microbiota (11%) (**Table 1**). These results varied significantly by country: antibiotic resistance was the greatest concern in South Korea (58%) and Indonesia (51%), possible side effects in India (59%) and lack of efficacy in Australia (36%) and Japan (52%).

**Table 1:** Risks associated with the prescription of an antibiotic as perceived by surveyed physicians.

	AUS N=100	JPN N=100	IDN N=90	IND N=140	CHN N=140	SGP N=67	KOR N=100	TOTAL N=737
Antibiotic resistance	37%	24% ↓	51% ↑	12% ↓	34%	26%	58% ↑	**34%**
Side effects	15% ↓	17% ↓	20%	59% ↑	21%	33%	22%	**27%**
Lack of efficacy	36% ↑	52% ↑	6% ↓	16% ↓	29%	29%	14% ↓	**26%**
Alteration of intestinal microbiota	12%	8%	14%	13%	15%	12%	6%	**11%**
None	0%	1%	9%	0%	1%	0%	0%	**2%**

Question B3: “Please rank by importance the potential risks that detract you from prescribing antibiotics to an adult”. Only top one is presented.

AUS, Australia; JPN, Japan; IDN, Indonesia; IND, India; CHN, China; SGP, Singapore; KOR, South Korea.

↑Significantly higher than other countries.

↓Significantly lower than other countries.

### Microbiota and Co-Prescription of Probiotics

Overall, a large majority of surveyed physicians agreed that antibiotics disrupted intestinal microbiota (85%) with a range from 69% in Indonesia to 94% in Japan and China. Rates for Australia, India, Singapore and Korea were 90%, 93%, 78% and 80%, respectively.

A probiotic was co-prescribed with an antibiotic in 27% of instances overall. Again, there were significant variations by country (**Figure 1**): from 16% and 20% in Japan and Australia, respectively, to 39% in Singapore.

**Figure 2:**
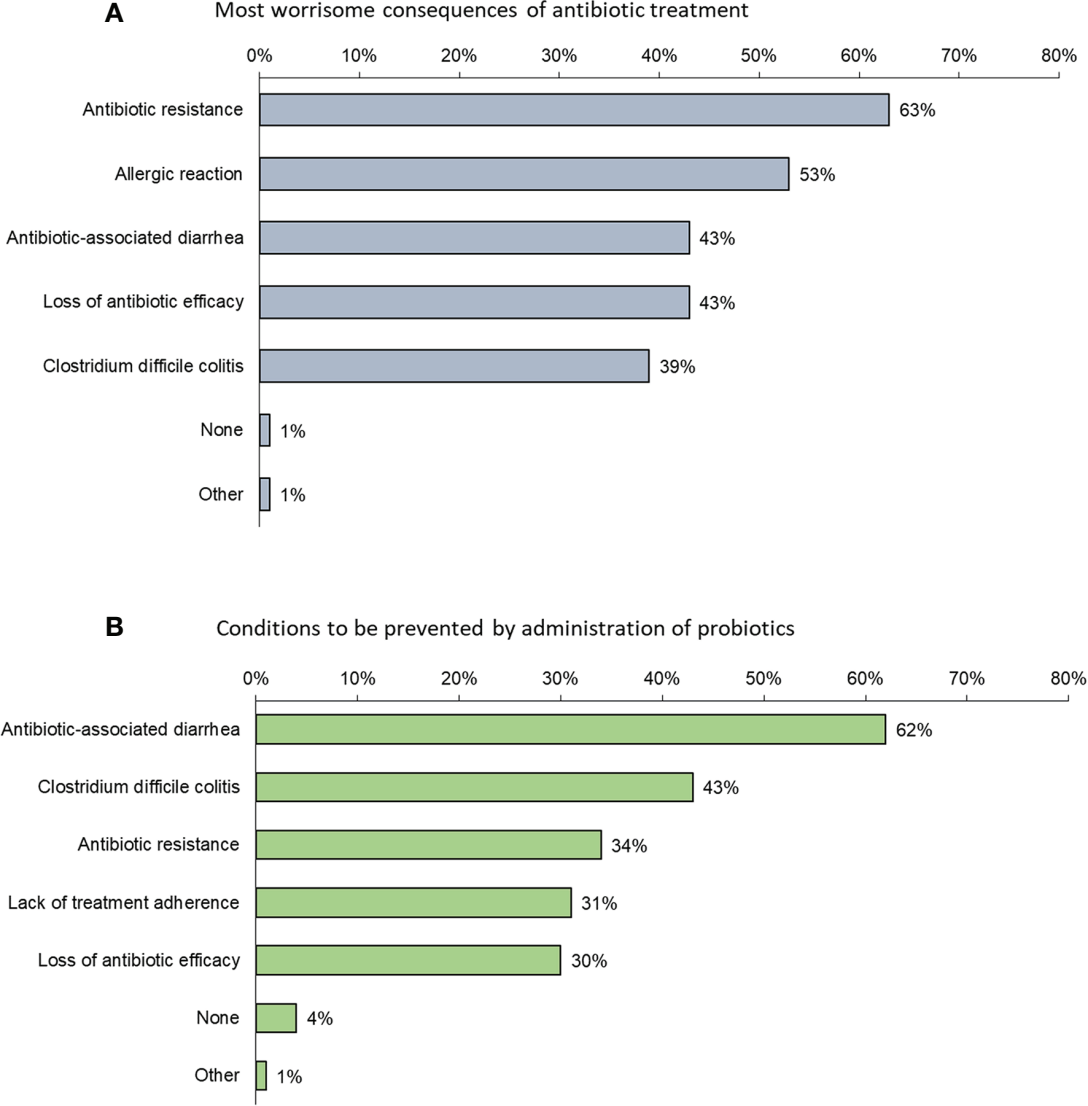
**(A)** Factors that most (in order of frequency) concern physicians when prescribing an antibiotic (Question B5: “*Among the following consequences of antibiotic treatment, which is/are the most worrisome to adults in your opinion?*”); **(B)** Conditions (in order of frequency) considered by physicians to be prevented by probiotics (Question B6: “*Which of the following conditions, in your opinion, may be prevented by the administration of probiotics to adults?*”).

When ranking what they perceived to be the most worrisome consequences of antibiotic treatment, physicians were most concerned about antibiotic resistance; overall, 63% ranked this as their greatest concern (ranged from 36% in India to 75% in Australia and 74% in China with rates for Japan, Indonesia, Singapore and Korea of 54%, 64%, 70% and 71%, respectively) (**Figure 2A**). Fear of an allergic reaction came second: 53% overall (91% in India, 45% in Australia, 43% in Japan, 49% in Indonesia, 51% in China, 47% in Singapore and 44% in Korea) and AAD came in third: 43% overall (52% in Australia, 43% in Japan, 35%, 39% in India, in Indonesia, 45% in China, 36% in Singapore, 54% in Korea).

Conditions considered by physicians to be prevented by probiotics were mostly AAD (62%) and *C. difficile* colitis (43%) (**Figure 2B**).

Patients who were most likely to be co-prescribed probiotics included adults with a history of previous episodes of AAD (56%), those receiving antibiotics with a perceived increased risk of diarrhea (e.g. amoxicillin/clavulanic acid) (53%), those requiring prolonged treatment with antibiotics (51%) and those who already had diarrhea (51%) (**Table 2**). Here again, significant between-country variations were observed. Physicians in Australia, for example, ranked a requirement for prolonged treatment with antibiotic highest (75%), followed by the use of antibiotics with a perceived increased risk of diarrhea (70%), previous episodes of AAD (70%) and risk of *C. difficile* infection (53%).

**Table 2:** Clinical factors that prompted the co-prescription of a probiotic with an antibiotic.

	AUS N=64	JPN N=81	IDN N=80	IND N=139	CHN N=136	SGP N=66	KOR N=95	Total N=661
Previous episodes of AAD	70% ↑	60%	51%	35% ↓	62%	49%	73% ↑	**56%**
AB with increased risk of diarrhea^a^	70% ↑	43%	29% ↓	65% ↑	62%	47%	60%	**53%**
Prolonged treatment with AB	75% ↑	42%	31% ↓	70% ↑	68% ↑	25% ↓	49%	**51%**
Presence of diarrhea	44%	62%	53%	21% ↓	60%	42%	77% ↑	**51%**
Underlying condition/comorbidities	64% ↑	22% ↓	34%	41%	48%	32%	51%	**41%**
Receiving broad-spectrum AB	50%	19% ↓	29% ↓	73% ↑	43%	32%	34%	**40%**
Older patients	44%	36%	29%	31%	49% ↑	42%	39%	**38%**
*C. difficile* infection	53% ↑	36%	12% ↓	31%	40%	46%	43%	**37%**
Malnutrition	31%	15% ↓	6% ↓	77% ↑	43%	39%	16% ↓	**34%**
Hospitalized patients	28%	7% ↓	34%	71% ↑	20% ↓	46% ↑	21% ↓	**33%**
Bloody diarrhea	22%	20%	27%	16%	22%	28%	27%	**23%**
Intravenous AB	28%	10% ↓	9% ↓	37% ↑	18%	29%	15%	**21%**
Other	0%	1%	4%	0%	1%	0%	0%	**1%**
Mean number of profiles quoted	5.8 ↑	3.7 ↓	3.5 ↓	5.7 ↑	5.4	4.6	5.0	**4.8**

Question B8: “For which type of adult patients receiving antibiotics, would you consider co-prescribing probiotics?” (base: at least 1% of patients co-prescribed with antibiotics)

AB, antibiotics; AUS, Australia; JPN, Japan; IDN, Indonesia; IND, India; CHN, China; SGP, Singapore; KOR, South Korea.

a e.g.,amoxicillin/clavulanic acid.

↑Significantly higher than other countries.

↓Significantly lower than other countries.

### Prescription of Antibiotics in Specific Clinical Situations

*1*. An adult patient with a 2-day history of acute respiratory symptoms and fever:

58% of gastroenterologists reported that they would provide symptom relief alone at the first visit and 48% chose to adopt a wait-and-see approach. 37% reported that they would prescribe an antibiotic at the first visit (**Table 3**).

*2*. An adult patient with a 2-day history of acute, presumed infectious diarrhea with fever:

56% of gastroenterologists reported that they would provide symptom relief alone at the first visit and 51% chose to adopt a wait-and-see approach. 41% reported that they would prescribe an antibiotic at the first visit (**Table 3**).

**Table 3:** Physician approaches to a clinical scenario involving either a 2-day history of acute respiratory symptoms (for GPs) or a 2-day history of acute infectious diarrhea and fever (for gastroenterologists).

	AUS	JPN	IDN	IND	CHN	SGP	KOR	TOTAL
2-day history of acute respiratory symptoms and fever^a^	**N=50**	**N=50**	**N=50**	**N=70**	**N=70**	**N=47**	**N=50**	**N=387**
Give medication for symptom relief	50%	36% ↓	56%	96% ↑	50%	66%	50%	**58%**
Adopt wait-and-see approach	70% ↑	8% ↓	72% ↑	59%	44%	60%	24% ↓	**48%**
Prescribe antibiotics (AB)	10% ↓	56% ↑	38%	21% ↓	31%	38%	62% ↑	**37%**
Other	6%	8%	2%	0%	11%	2%	4%	**5%**
Prescribe nothing	8% ↑	8% ↑	0%	0%	0%	0%	0%	**2%**
Patients expecting AB prescription^b^	44%	43%	52%	25% ↓	59% ↑	43%	46%	**44%**
2-day history of acute infectious diarrhea and fever^c^	**N=50**	**N=50**	**N=40**	**N=70**	**N=70**	**N=20**	**N=50**	**N=350**
Give medication for symptom relief	22% ↓	44%	52%	97% ↑	49%	85% ↑	40% ↓	**56%**
Adopt wait-and-see approach	78% ↑	4% ↓	80% ↑	74% ↑	39%	45%	34% ↓	**51%**
Prescribe antibiotics (AB)	2% ↓	62% ↑	28%	16% ↓	49%	75% ↑	54%	**41%**
Other	16% ↑	8%	5%	0%	6%	5%	8%	**7%**
Prescribe nothing	6% ↑	6% ↑	0%	0%	0%	0%	0%	**2%**
Patients expecting AB prescription^d^	39%	45%	42%	29% ↓	62% ↑	39%	40%	**42%**

AUS, Australia; JPN, Japan; IDN, Indonesia; IND, India; CHN, China; SGP, Singapore; KOR, South Korea.

a Question B10: “You see an adult patient with a 2-day history of acute respiratory symptoms and fever. The patient request antibiotics. You would…”.

b Question B11: “In your practice, what percentage of adult patients expect an antibiotic prescription during their first visit for an acute respiratory infection and fever?”.

c Question B14: “You see an adult patient with a 2-day history of acute infectious diarrhea and fever. The parents request antibiotics. You would…”.

d Question B15: “In your practice, what percentage of adult patients expect an antibiotic prescription during their first visit for acute infectious diarrhea and fever?”.

↑Significantly higher than other countries.

↓Significantly lower than other countries.

Overall, an antibiotic prescription was expected at the first visit by 44% of patients with acute respiratory symptoms with fever and by 42% with acute infectious diarrhea with fever (**Table 3**). There was no relationship between the rates of patients’ expectation of antibiotics and the rates of antibiotic prescription. Thus, the highest rates of patients’ expectation of antibiotics were reported in China (59% and 62% for the two conditions, respectively), but the rates of antibiotic prescription remained relatively low (31% and 49%). The lowest rates for patients’ expectation of antibiotics were for India (25% and 29%, respectively) where actual rates of antibiotic prescription were also lowest (21% and 16%, respectively). The lowest antibiotic prescription rates were in Australia at 10% and 2%, respectively.

**Figure 3:**
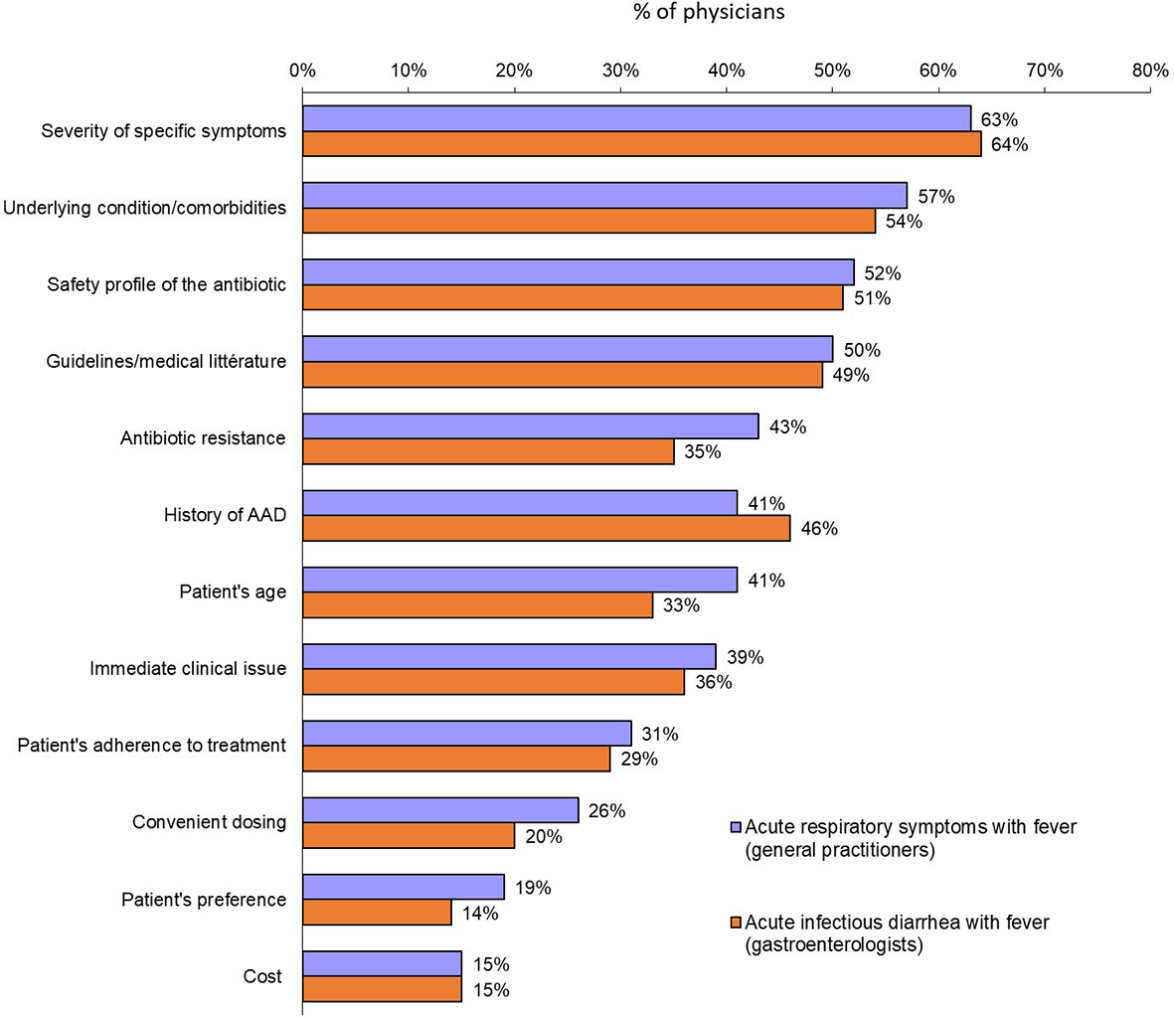
Factors most likely to prompt a physician to prescribe an antibiotic for either acute respiratory symptoms with fever (general practitioners) or acute infectious diarrhea with fever (gastroenterologists) (Question B12: “*When deciding to prescribe an antibiotic therapy for acute respiratory symptoms with fever to an adult, which one of the following factor(s) will you consider?*”: Question B16: “*When deciding to prescribe an antibiotic therapy for acute infectious diarrhea with fever to an adult, which one of the following factor(s) will you consider?*”).

The clinical factors that predicted a physician opting for an antibiotic prescription were similar for both scenarios: symptom severity (63% and 64%, for acute respiratory illness and acute diarrhea, respectively), presence of significant underlying condition/comorbidities (57% and 54%) and safety profile of the antibiotic (52% and 51%) (**Figure 3**). There were some significant differences according to countries. Thus, for patients with acute respiratory symptoms with fever, history of AAD was considered for prescribing or not antibiotics by 76% of GPs in India and only 24% in Japan and Indonesia. The safety profile of the antibiotic was an important factor for Chinese GPs and gastroenterologists (84% and 83%, respectively).

For patients with acute respiratory symptoms with fever, the most frequent class chosen by GPs was beta-lactams (55%) followed by fluoroquinolones (21%) and macrolides (15%). In Japan, the order was macrolides (42%) followed by fluoroquinolones (30%) and beta-lactams (22%).

For patients with acute infectious diarrhea with fever, the most frequent antibiotic class chosen by gastroenterologists was that of fluoroquinolones (45%) followed by beta-lactams (30%) and macrolides (13%). For Indonesia and India, the order was beta-lactams (45% and 59%, respectively) followed by fluoroquinolones (25% and 14%) and co-trimoxazoles (17% for both) (For details of antibiotics used see **Supplementary Table 1**).

## Discussion

This survey on attitudes of physicians toward prescription of antibiotics and co-prescription of probiotics was performed in seven countries of the Asia-Pacific region. Among them three are considered as low- or middle-income countries (India, Indonesia and China) and four high-income countries (Australia, Japan, South Korea and Singapore), according to the International Bank for Reconstruction and Development. Moreover, India, Indonesia and China are listed among the 15 countries with the largest burden of pneumonia and diarrhea deaths (International Vaccine Access Center, 2018).

**Figure 4:**
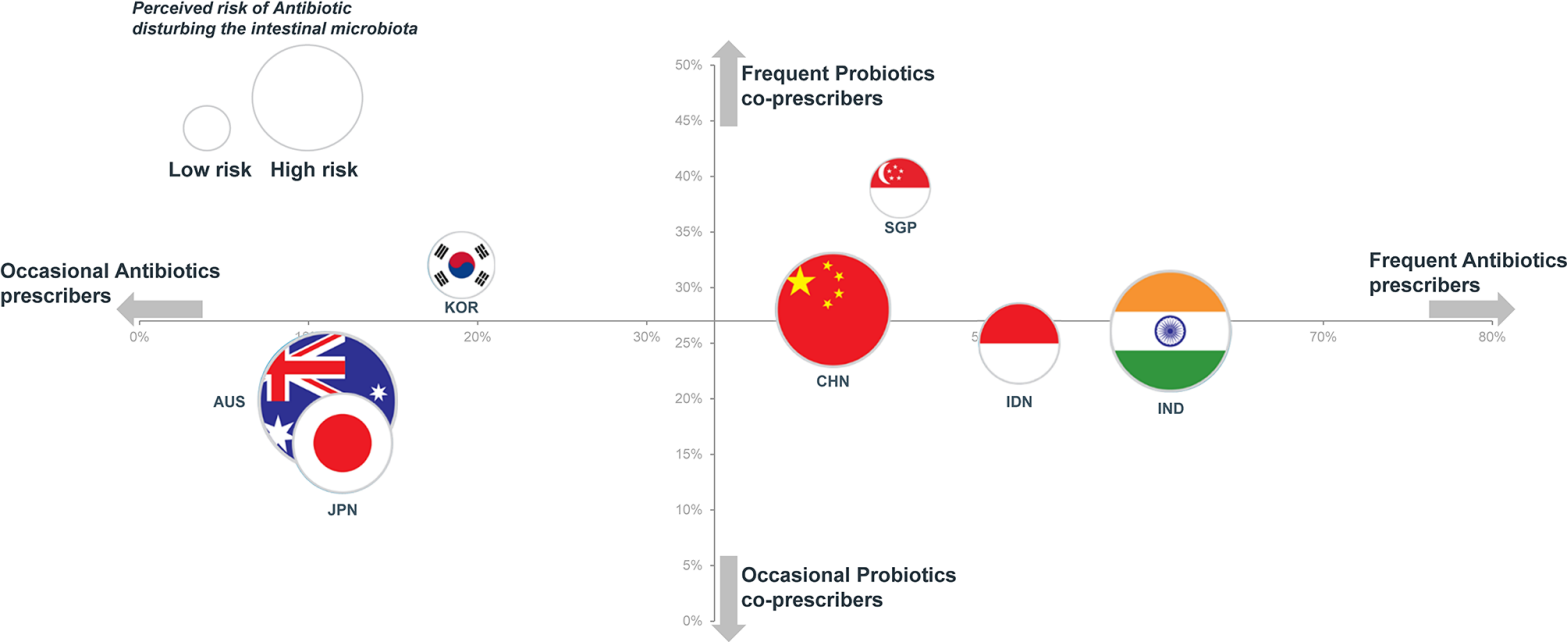
Rates of antibiotic prescription (x-axis) vs. probiotics prescription (y-axis) and perceived risk of antibiotic disturbing the intestinal microbiota (flag surface area).

Attitudes towards the use of antibiotics and probiotics varied considerably by country. The relationship between the rates of prescriptions of antibiotics and probiotics is depicted on **Figure 4**. The seven countries appeared to be distributed in two clusters. The first cluster included Australia, Japan and South Korea, which are low antibiotic prescribers (from 11% to 19% of physician visits) and high-income countries. In line with their overall low rates of antibiotic prescription, Australian physicians were more likely to adopt a wait-and-see approach to either acute respiratory symptoms (70%) or acute infectious diarrhea (78%) and, accordingly, were least likely to prescribe an antibiotic at the first visit (10% for acute respiratory symptoms and 2% for acute infectious diarrhea).

Although Japan was included in the cluster of occasional antibiotic prescribers, the antibiotic prescription option was more frequently chosen (56%) in the acute respiratory symptoms with fever scenario with a wait-and-see approach being chosen by only 8% of GPs. Similarly, for an acute infectious diarrhea with fever, antibiotics were highly favored by gastroenterologists in Japan (62%) with the adoption of a wait-and-see approach being very rare (4%). Similar responses to these clinical scenarios were observed among physicians in South Korea.

In contrast to other high-income countries physicians in Singapore were frequent prescribers of antibiotics (45%) and probiotics (39%). This is perhaps related to the fact that physicians in Singapore might also act as pharmacists and sell medications to their patients.

The second cluster described in **Figure 4** included China, Singapore, Indonesia and India, which are high antibiotic prescribers (from 41% to 61% of visits). With the exception of Singapore, these are also among those countries with the largest burden of pneumonia and diarrhea deaths. Although Indonesia and India were in the cluster of high antibiotic prescribers, physicians of these countries were less likely to opt for an antibiotic prescription as the initial strategy for the patient with a 2-day history of acute infectious diarrhea and fever (28% and 16%, respectively); instead they were most likely to adopt a wait-and-see approach (80% and 74%) (**Table 3**). Comparable attitudes were reported by GPs in Indonesia and India for the scenario involving acute respiratory symptoms with fever.

According to the responses of both GPs and gastroenterologists from that country, an expectation of a prescription for antibiotics appeared highest in China (59% for acute respiratory symptoms and 62% for acute infectious diarrhea).

We note in **Figure 4** that no clear relationship can be established between the perceived risk of antibiotics disturbing the intestinal microbiota and actual rates of prescription of either antibiotics or probiotics. While concerns regarding the potential impact of antibiotics on the gut microbiota were widely shared, this does not seem to lead to the co-prescription of probiotics. Thus, 90% of physicians in Australia agreed that antibiotics disrupted intestinal microbiota, but only 20% co-prescribed probiotics. In contrast to concerns surrounding the development of antibiotic resistance, the possibility of an alteration of intestinal microbiota was ranked low when physicians were asked to rank their perceived risks relating to the prescription of an antibiotic (**Table 1**). Similarly, 94% of physicians in Japan felt that antibiotics disrupted intestinal microbiota, but only 16% considered that AAD could be prevented by the co-administration of probiotics (this contrasts with an average of 62% for all surveyed physicians) (**Figure 2**).

Overall, the attitudes of physicians regarding the prescription of antibiotics and co-prescription of probiotics appeared to vary greatly between countries. The burden of infectious disease in a given country, together with variations in access to drugs and differences in health care delivery could explain, at least in part, these differences (Klein et al., 2018). Variations in understanding on antibiotic use, the gut microbiota and probiotics may also be relevant. Here, education, not only to prescribers but also patients, on the prudent use of antibiotics could have a very positive impact. Educational programs that aimed to correct misunderstandings about antibiotic use have been shown to be effective in reducing their use (Lee et al., 2015). The 2016–2018 WHO Report on Surveillance of Antibiotic Consumption underscored the need to ensure the appropriate use of antibiotics through prescription-only policies and the implementation of antimicrobial stewardship programs.

This study has all of the limitations of a declarative survey. Thus, data were not obtained from real patient files, but reflected the perception of physicians about their own practice. As a consequence, there were some apparent discrepancies in some countries between the rates at which physicians actually prescribed antibiotics in their practices and the choices they made in response to the clinical scenarios. Furthermore, for some countries, the sample size of some physician groups (e.g. gastroenterologists in Singapore) was low, precluding complete analysis. In large countries like China, the answers to the survey may not be representative of the diversity of the medical practices because the surveyed physicians were mainly urban and practicing in public hospitals.

## Conclusions

According to this online survey in GPs and gastroenterologists in the Asia-Pacific area, the co-prescription of probiotics is low in many countries. Nevertheless, physicians are aware of the adverse effects of antibiotics on intestinal microbiota and the benefits of probiotics in preventing them.

## Data Availability Statement

The raw data supporting the conclusions of this article will be made available by the authors, without undue reservation.

## Author Contributions

Conceptualization, UG, K-AG, GH, YL, SP, MS, HC, and EQ. Data curation, KS. Formal analysis, UG, HC, and EQ. Methodology, UG, K-AG, GH, YL, SP, MS, KS, HC, and EQ. Supervision, UG, HC, and EQ. Writing – original draft, UG, HC, and EQ. Writing – review & editing, UG, K-AG, GH, YL, SP, MS, KS, HC, and EQ. All authors contributed to the article and approved the submitted version.

## Funding

This work was funded by Biocodex.

## Supplementary Material

The Supplementary Material for this article can be found online at: https://www.frontiersin.org/articles/10.3389/fcimb.2021.722700/full#supplementary-material

## Conflict of Interest

EQ reports personal fees from Alimentary Health, Biocodex, Salix, Menarini, grants from 4D Pharma and Vibrant, and other from Alimentary Health, outside the submitted work.

The remaining authors declare that the research was conducted in the absence of any commercial or financial relationships that could be construed as a potential conflict of interest.

## Publisher’s Note

All claims expressed in this article are solely those of the authors and do not necessarily represent those of their affiliated organizations, or those of the publisher, the editors and the reviewers. Any product that may be evaluated in this article, or claim that may be made by its manufacturer, is not guaranteed or endorsed by the publisher.
