# Supplementary material for: Physician Perceptions on the Use of Antibiotics and Probiotics in Adults: An International Survey in the Asia-Pacific Area
Source: Front Cell Infect Microbiol. 2021 Oct 19;11:722700. doi: 10.3389/fcimb.2021.722700 (PMC8562691; doi:10.3389/fcimb.2021.722700)
Supplement: Supplementary file 1 [file Table_1.docx]

|  | **Australia** | **China** | **India** | **Indonesia** | **Japan** | **Korea** | **Singapore** |
| --- | --- | --- | --- | --- | --- | --- | --- |
| **Acute Respiratory Symptoms with fever** | Beta-lactam (78%) | Beta-lactam (59%)  Fluoroquinolone (36%) | Beta-lactam (69%) | Beta-lactam (69%) | Macrolide (42%)  Fluoroquinolone (30%) | Beta-lactam (78%) | Beta-lactam (38%)  Fluoroquinolone (26%) |
| **Acute Infectious Diarrhea with fever** | Fluoroquinolone (68%) | Fluoroquinolone (60%) | Beta-lactam (59%) | Beat-lactam (45%)  Fluoroquinolone (25%) | Fluoroquinolone (66%) | Fluoroquinolone(66%) | Beta-lactam (50%)  Macrolide (35%) |

**Supplementary Table 1.** Most frequently prescribed antibiotics by country and for each indication. Numbers in parentheses refer to the percentage of responders who chose that particular antibiotic.
